# Dose-dependent seizure control with MEK inhibitor therapy for progressive glioma in a child with neurofibromatosis type 1

**DOI:** 10.1007/s00381-022-05571-y

**Published:** 2022-06-01

**Authors:** Evan Cantor, Ashley Meyer, Stephanie M. Morris, Judith L. Z. Weisenberg, Nicole M. Brossier

**Affiliations:** 1 Department of Pediatrics, Washington University School of Medicine, 660 South Euclid Avenue, Box 8208, St. Louis, MO 63110, USA; 2 Department of Neurology, Washington University School of Medicine, St. Louis MO, USA

**Keywords:** Epilepsy, Seizure, Neurofibromatosis, NF1, MEK inhibitor, Selumetinib

## Abstract

**Background:**

Low-grade gliomas (LGGs) occurring in children can result in many different neurologic complications, including seizures. MEK inhibitors are increasingly being used to treat LGG, but their effect on associated neurologic symptoms has not been established.

**Results:**

Here, we report a patient with neurofibromatosis type 1 (NF1), medically refractory epilepsy (MRE), and an extensive optic pathway glioma (OPG) who developed dose-dependent seizure control while being treated with selumetinib. Seizure frequency rebounded after dose reduction for cardiac toxicity, then improved, and finally ceased after restarting full dosing, allowing confidence in the cause of improvement.

**Conclusion:**

Selumetinib may have promise in epilepsy management in other children with NF1 or LGG.

## Introduction

LGGs are common tumors in childhood and can result in neurologic complications, including seizures [1, 2]. In the following case, we describe a patient who achieved dose-dependent seizure control while on MEK inhibition for his NF1-associated OPG, with re-emergence of seizures after dose reduction and subsequent resolution of seizures upon return to full dose.

## Results

**Fig. 1:**
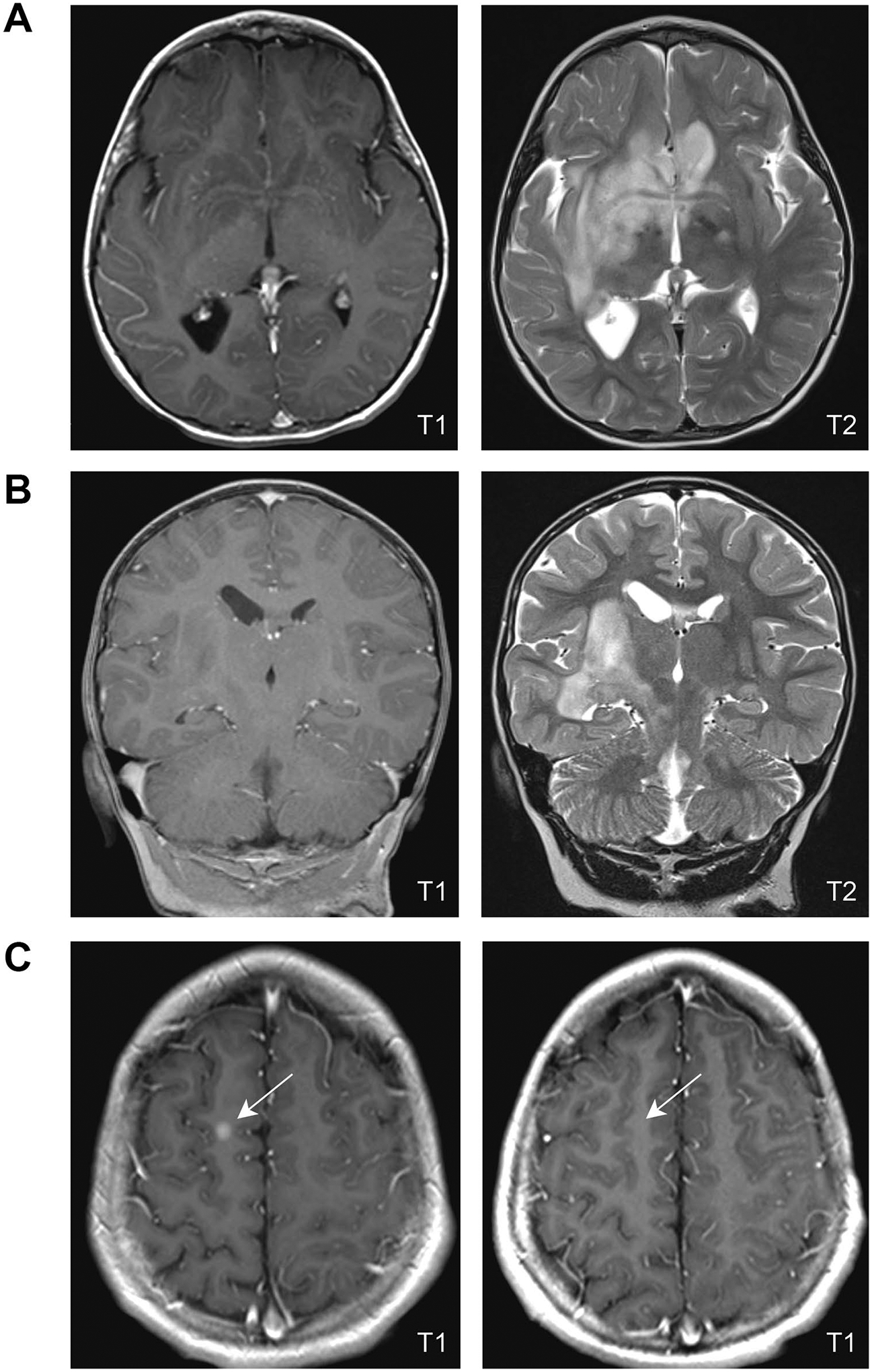
Imaging characteristics at initial MRI. **A** Axial images from the patient’s original MRI are shown, illustrating the T1 hypointense (**A**, left panel) and T2 hyperintense (**A**, right panel) expansile lesions outside the optic pathway. **B** Coronal imaging of the mesial temporal lobe at diagnosis (T1, left panel; T2, right panel). **C** Post-contrast axial T1 sequences show enhancing nodule in right frontal lobe prior to therapy initiation (**C**, left panel) that resolved with treatment (**C**, right panel)

The patient was brought to medical attention at age 3 due to spells concerning for possible seizures. Physical examination was notable for café-au-lait macules, axillary freckling, and left-sided facial weakness. Routine awake EEG revealed focal epileptiform discharges and prominent localized slowing in the right temporal region. MRI of the brain revealed bilateral OPG with extension into the hypothalamus, basal ganglia, and subcortical white matter (Fig. 1A), and mass effect on the right insular cortex and abnormal hippocampal signal intensity (Fig. 1B). He was diagnosed with NF1 and focal symptomatic epilepsy. He was started on oxcarbazepine and referred to Pediatric Neuro-Oncology, who recommended treatment if he progressed.

He re-established care with Pediatric Neuro-Oncology at 8 years old, while under consideration for vagal nerve stimulator implantation for MRE. At that time, seizures occurred in clusters every 5–6 days, with seizure frequency ranging between 1 and 10 seizures per day for 3–5 days during clusters, despite management with clobazam 0.3 mg/kg/day, levetiracetam 58 mg/kg/day, and CBD oil. A 24-h EEG captured three electroclinical seizures that lateralized to the right hemisphere but were poorly localized. Repeat brain MRI revealed slow progression of his glioma over the prior 2 years with a new area of enhancement in his right frontal lobe (Fig. 1C, left). Examination was notable for dysarthria, left lower facial weakness, left upper extremity weakness, and mild circumduction of left lower extremity while walking. His mother also reported concerns regarding academic performance despite having an individualized education plan in place. Family refused standard of care therapy with carboplatin and vincristine, but agreed to MEK inhibitor (MEKi) treatment. The patient was started on 25 mg/m^2^ selumetinib BID.

After 12 weeks, the patient’s mother noted significantly decreased seizure frequency with improved academic performance. Repeat brain MRI showed stable mass size but interval reduction in enhancement of the right frontal lesion (Fig. 1C, right), suggestive of treatment response. Unfortunately, he displayed evidence of cardiac toxicity on routine evaluation at this time (asymptomatic decline in left ventricular ejection fraction, 63% to 47%). Selumetinib was held, then reinitiated at 50% dosing upon subsequent normalization of echocardiogram findings.

On dose-reduced selumetinib, the patient’s mother reported that seizure frequency increased to pre-treatment baseline. Given the re-emergence of poorly controlled seizures, the family and treatment team both aimed to resume 100% dosing. He was started on afterload reduction with enalapril for cardioprotection and escalated back to 100% selumetinib dosing the following month.

Four weeks after restarting on full dose, the patient’s mother again reported a drastic improvement of both seizure frequency and academic performance. Improvements in alertness, left-sided weakness, and gait were also noted on neurologic examination. Currently, the patient remains seizure-free on full-dose selumetinib with stable disease on brain MRI. A recent 24-h EEG captured no electrographic or clinical seizures.

## Discussion

### NF1 and epilepsy

In addition to the propensity to develop nervous system tumors, patients with NF1 have an increased risk of epilepsy, with 4–9.5% of patients developing seizures vs 1–2% in the general population [3, 4]. Focal sources — most commonly CNS tumors or mesial temporal sclerosis [4] — are frequently identified in NF1 patients with unprovoked seizures [3, 4], with 75% of patients demonstrating lateralizing epileptiform foci on EEG [3].

While children with NF1 are at increased risk of developing LGGs, these typically arise in the optic pathway [5] or brainstem [6], locations usually not associated with seizures. In contrast, this particular case may be more illustrative of the recently described “deep extensive gliomas” (DEGs) in children with NF1, which involve bilateral temporal lobes, basal ganglia, and thalami in addition to the optic pathway and may have a more severe clinical course [7]. Thirty-three percent of reported DEGs were associated with epilepsy [7]. It is unclear if standard treatment will improve seizure control in these cases, although decreased seizure frequency has been observed with treatment of some sporadic LGGs [8].

Importantly, not all patients with NF1-associated epilepsy have focal findings on imaging or EEG [3, 4], suggesting that genetic mutation alone may predispose to the development of seizure. In support of this hypothesis, patients with other genetic syndromes resulting in increased MAPK pathway activation, such as Noonen’s [9], tuberous sclerosis (TSC) [10, 11], or SYNGAP1 deficiency [12], have an increased incidence of epilepsy [11, 13, 14]. MAPK signaling has also been implicated in epilepsy outside of the context of germline mutation — microarray data from hippocampi of temporal lobe epilepsy patients shows differential regulation of genes within the MAPK pathway [15], and increased ERK activation is observed in murine hippocampal tissue at the time of spontaneous seizure [16]. In addition, BRAF V600E mutation in early brain development (as hypothesized to occur in sporadic pediatric LGG) results in both mutant neurons and glia in murine models; mutation induces epileptogenic changes in the former [2]. Although this patient’s seizures were clearly focal in origin, it is possible that MAPK activation due to *NF1* germline mutation in neuronal cells might contribute to the severity of his epilepsy and predict response to MEK inhibition. Notably, MEK inhibition has resulted in reduced seizure activity in mouse models of TSC [11] and in the Krushinsky–Molodkina rat [17], a genetic rat model of audiogenic seizures in which increased ERK activation is observed in glutamatergic neurons [18].

### Potential mechanisms of action

While the mechanism by which MEK inhibition might decrease epileptogenic activity has not been established, abnormalities of both glutamatergic and GABAergic pathways have been identified in MAPK-activated murine models. For example, expression of a constitutively active MEK1 (caMEK1) mutant in the murine brain results in spontaneous seizures, a phenotype dependent on increased eIF4E-mediated translation of the NMDA glutamate receptor NR2B subunit downstream of activated ERK in neurons [19]. When specifically targeted to GABAergic-interneurons, caMEK1 leads to spontaneous epileptiform activity accompanied by reduced inhibitory synapses on excitatory glutamatergic neurons [20]. Together, this suggests that MEKi may decrease aberrant neuronal excitability in NF1 by reducing glutamatergic stimulation and/or increasing GABAergic signaling.

### Biologically targeted agents for treatment for epilepsy

To our knowledge, this case is the first to describe the effective treatment of epilepsy with a MEKi in a human patient. This may be in part because therapeutic trials of MEKi efficacy in LGGs have thus far excluded patients with MRE [21, 22]. Frequent adjustments of AEDs may also make attribution of response difficult. Notably, in this case, a dose-dependent response to MEK inhibition was observed without concurrent changes in AEDs, allowing us to be more confident in the cause of improvement. Importantly, this patient’s tumor was stable in size despite decreased enhancement with treatment, suggesting that objective response by RANO criteria [23] is not necessary for improved seizure control.

## Conclusion

In this case, our patient appeared to have dose-dependent seizure control while receiving MEK inhibition for his glioma. This suggests that MEKi may be beneficial in the treatment of seizures in other children with brain tumors or with genetic disorders affecting the RAS/MAPK pathway. Further data from multiple patients with confirmatory EEG and neuropsychosocial testing will be needed to confirm these findings.

## Abbreviations

LGG: Low-grade glioma
NF1: Neurofibromatosis type 1
OPG: Optic pathway glioma
EEG: Electroencephalogram
MRI: Magnetic resonance imaging
MRE: Medically refractory epilepsy
AED: Anti-epileptic drug
CBD: Cannabidiol
MEKi: MEK inhibitor
DEG: Deep extensive glioma
